# Saturated Fatty Acids and Cardiovascular Disease: Replacements for Saturated Fat to Reduce Cardiovascular Risk

**DOI:** 10.3390/healthcare5020029

**Published:** 2017-06-21

**Authors:** Michelle A. Briggs, Kristina S. Petersen, Penny M. Kris-Etherton

**Affiliations:** 1 Department of Biology, Lycoming College, 700 College Place, Williamsport, PA 17701, USA; 2 Department of Nutritional Sciences, The Pennsylvania State University, University Park, PA 16802, USA; kup63@psu.edu (K.S.P.); pmk3@psu.edu (P.M.K.-E.)

**Keywords:** dietary substitution, CVD, saturated fatty acids, protein, monounsaturated fatty acids, polyunsaturated fatty acids, dairy fat, refined carbohydrates, whole grains

## Abstract

Dietary recommendations to decrease the risk of cardiovascular disease (CVD) have focused on reducing intake of saturated fatty acids (SFA) for more than 50 years. While the 2015–2020 Dietary Guidelines for Americans advise substituting both monounsaturated and polyunsaturated fatty acids for SFA, evidence supports other nutrient substitutions that will also reduce CVD risk. For example, replacing SFA with whole grains, but not refined carbohydrates, reduces CVD risk. Replacing SFA with protein, especially plant protein, may also reduce CVD risk. While dairy fat (milk, cheese) is associated with a slightly lower CVD risk compared to meat, dairy fat results in a significantly greater CVD risk relative to unsaturated fatty acids. As research continues, we will refine our understanding of dietary patterns associated with lower CVD risk.

## 1. Introduction

Cardiovascular disease (CVD) is the number-one cause of death and disability worldwide [1]. Poor diet, moreover, is a leading risk factor for CVD [1,2]. Therefore, dietary improvements have the potential to significantly reduce the prevalence of CVD [3]. It is well established that saturated fatty acids (SFA) increase low-density lipoprotein (LDL) cholesterol, a strong risk factor for CVD [4]. The 2013 American Heart Association and American College of Cardiology (AHA/ACC) Guideline on Lifestyle Management to Reduce Cardiovascular Risk reports strong evidence (level A) for reducing SFA intake (5% to 6% of calories) to lower LDL cholesterol [5]. Similarly, The National Lipid Association Expert Panel strongly recommends (Grade A evidence) a diet low in SFA (<7% of energy) [6]. Despite this strong evidence to limit SFA, current intake in the USA is 10.7% of energy [7]. Recently, however, some meta-analyses of observational studies have shown no association between SFA and CVD risk [8,9]. These studies have led to questions about whether dietary SFA should be restricted, as recommended by authoritative federal agencies and professional societies. It must be acknowledged, though, that many of these analyses did not take into consideration the dietary replacement of SFA. It is known that the observed effect will vary based on the foods and nutrients that replace SFA. The aim of this paper is to review the epidemiological and interventional evidence for the cardiovascular impact of replacements for SFA including MUFA, PUFA, *n*-*3* and *n*-*6* PUFA, protein, and carbohydrate including refined and whole grain sources. Replacement of SFA with *trans*-fat has not been considered because it is well established that *trans*-fat increases CVD risk and authoritative bodies recommend limiting intake and therefore it is not an appropriate replacement for SFA.

Traditionally, nutrition research has focused on single nutrients, however it is now recognized that the total diet must be considered because of the interdependent relationships among dietary components [10]. In the case of SFA, where the key feature of guidelines for CVD prevention is reducing intake, it is important to consider which foods and/or nutrient replacements are associated with a reduction in CVD risk. The AHA/ACC Guideline on Lifestyle Management to Reduce Cardiovascular Risk does not specify what macronutrient should replace SFA, but states more favorable effects on lipid profiles are observed when SFA is replaced by PUFA, followed by MUFA, then carbohydrates [5]. The National Lipid Association Expert Panel also states that replacing SFA with unsaturated fats, proteins, or carbohydrates lowers levels of atherogenic cholesterol but replacement with unsaturated fat or protein elicits greater reductions than carbohydrates [6]. These authoritative bodies do not set guidelines for intake of total fat, MUFA, PUFA, protein or carbohydrates. The Institute of Medicine sets Acceptable Macronutrient Distribution Ranges (AMDR), defined as a range of intake that is associated with a reduced risk of chronic disease while providing adequate intake of essential nutrients [11]. The AMDRs for total fat, carbohydrate and protein are 20–35%, 45–65% and 10–35% of total energy, respectively. The AMDRs for *n*-*6* PUFA, and α-linolenic acid are 5–10% of energy and 0.6–1.2% of energy, respectively. Approximately 10% of the AMDR for α-linolenic acid can be consumed as Eicosapentaenoic Acid (EPA) and/or Docosahexaenoic Acid (DHA) (0.06–0.12% of energy). An AMDR is not set for MUFA since they are not essential. 

## 2. SFA

### 2.1. Short, Medium and Long Chain SFA & CVD Risk

Medium chain fatty acids (MCFA) contain 7–12 saturated carbons, while short chain fatty acids contain 1-6 saturated carbons [12]. Long chain fatty acids (LCFA) contain 13 or more carbons that can either be saturated or contain one or more double bonds. These structural variations lead to differences in absorption, transport and even destination [13]. For example, MCFA are absorbed from the gastrointestinal tract more efficiently than LCFA, and are transported via the portal vein directly to the liver for rapid oxidation, while LCFA are packaged into chylomicrons and travel through the lymphatic system, allowing for greater uptake by adipose tissue. Upon entering cells, MCFA can move into mitochondria without the carnitine shuttle, and appear to preferentially undergo fatty acid oxidation. LCFA, however, require the carnitine shuttle for transport into mitochondria [14]. When MCFA replace long chain triglycerides in the diet, these different metabolic routes appear to promote satiety and increase energy expenditure, possibly leading to weight control [14]. 

With regard to CVD, there are currently few clinical studies that have examined the effects of MCFA on CVD risk factors, and the results of these studies are inconsistent (reviewed by Kris-Etherton and Fleming [15]). In the Nurse’s Health Study (NHS) no significant increase in coronary heart disease (CHD) risk was associated with consuming short to medium chain SFA (4:0 to 10:0; *p* ≥ 0.60), although consuming greater amounts of longer chain SFA (sum of 12:0–18:0) increased CHD risk relative to the lowest intake group (relative risk (RR) 1.14; 95% confidence interval (95% CI) 0.93; 1.39, *p* = 0.03 after adjustment) [16]. A more recent analysis that included the NHS and Health Professionals Follow-Up Study (HPFS) confirmed earlier findings, and showed that longer chain SFA (12:0–18:0) increased CHD risk [17]. Lauric acid (12:0) is the fatty acid that increases LDL cholesterol to the greatest extent. However, it also has the greatest high-density lipoprotein (HDL) cholesterol raising effect, and therefore decreases the total cholesterol:HDL cholesterol ratio [18,19]. MCFA are common in coconut oil, palm kernel oil and dairy products, sources that also are rich in long chain SFA.

The most common saturated LCFA in the American diet are myristic acid (14:0), palmitic acid (16:0) and stearic acid (18:0). There is a great deal of overlap in their typical food sources. For example, dietary sources of myristic acid include palm kernel oil, coconut oil and butter, while dietary sources of palmitic acid include palm kernel oil, dairy fat, meats, cocoa butter, soybean and sunflower oils. Myristic and palmitic acids have comparable effects on both LDL and HDL cholesterol, but overall have little effect on the total cholesterol: HDL cholesterol ratio [19]. Stearic acid, compared with other SFA, has been shown to lower plasma LDL cholesterol levels, and have no effect on HDL cholesterol [20,21]. Therefore, even though stearic acid is a SFA, it does not appear to adversely affect CVD risk, possibly because it is desaturated in part to oleate (18:1 *n*-*9*) during metabolism [22]. Of note is that current treatment guidelines and recommendations target LDL cholesterol and non-HDL cholesterol for reduction to decrease CVD risk, because the benefits of also increasing HDL cholesterol are not entirely clear [23,24].

### 2.2. Dairy Fatty Acids

#### 2.2.1. The Association Between Dairy Fat and CVD Risk

Cheese (16.5% of SFA intake) and milk (8.3% of SFA intake) are among the top sources of SFA in the U.S. diet [25]. Fat-free or low-fat dairy are recommended in the current Dietary Guidelines for Americans (2015–2020) [26]. However, based on a number of recent studies, mass media reports are suggesting that full fat dairy is better for consumers. Rice reviewed 18 epidemiological studies that showed total dairy intake did not contribute to higher CVD risk, and that consuming milk or fermented dairy products such as yogurt and cheese may reduce CVD risk [27]. As Rice noted, the studies reviewed did not always specify how much of each food was consumed, thus limiting the translation of findings to consumer messaging. Further, even if the studies quantified consumption amounts, there was marked inconsistency between what one study defined as high intake compared to another study. 

Other studies have found similar results. A meta-analysis of 26 studies again found milk, cheese, and combined dairy were not associated with CVD mortality, although there was a high degree of heterogeneity between studies, and the studies available for analysis were of low quality [28]. In another analysis, Kratz et al., reviewed 16 studies that examined the association of full fat dairy consumption with obesity [29]. Of these, eleven studies found that consuming more dairy fat was associated with less weight gain over time compared to consuming less dairy fat. None of the 16 studies found that low-fat dairy was associated with an increased obesity risk, although the authors cautioned that some studies were flawed in their analyses. 

A meta-analysis of prospective cohort studies examining the association between butter consumption and the risk of CVD showed that when studies were normalized to an intake of one tablespoon a day (14 g), butter was borderline weakly associated with all-cause mortality (*n* = 9 studies, RR 1.01, 95% CI 1.00, 1.03, *p* = 0.045), but not associated with CVD (*n* = 4 studies, RR 1.00, 95% CI 0.98, 1.02, *p* = 0.704), CHD (*n* = 3 studies, RR 0.99, 95% CI 0.96, 1.03, *p* = 0.537) or stroke (*n* = 3 studies, RR 1.01, 95% CI 0.98, 1.03, *p* = 0.737) [30]. The authors concluded that relatively small or neutral associations exist between butter, mortality and CVD, indicating that butter should not be recommended for CVD prevention. A tablespoon (14 g) is a relatively small quantity but it calorically represents 5% of a 2000-calorie diet and 35% of the recommended intake of SFA. Similarly, Praagman et al.’s analysis of data from the European Prospective Investigation into Cancer and Nutrition (EPIC)-Netherlands cohort indicated that greater consumption of SFA from dairy sources including butter and cheese was associated with significantly lower risk of ischemic heart disease (IHD) [31]. It is important to note that neither of these studies investigated what foods participants consumed (e.g., refined carbohydrates or SFA from meat sources) when butter intake was low; this likely explains the different results observed in the studies by Pimpin et al., [30] and Praagman et al., [31]. Additionally, in the EPIC-Netherlands study [31], the authors indicated there was little variation in SFA intake within the population, and that *trans*-fat or cholesterol-lowering medications may have impacted their findings.

While the impact of full fat dairy products is difficult to determine based on the issues noted above, dairy products contain other compounds that may reduce CVD risk. In whole or reduced fat milk and yogurt products, fats are consumed along with minerals such as calcium, phosphorous, magnesium and potassium, elements that have blood pressure lowering effects [27]. There is some evidence that the calcium present in dairy products may attenuate the effect of dairy fats on blood lipid levels. In a four-way crossover study consumption of a low calcium, high fat diet (25% of energy from SFA; 474 mg calcium) significantly increased total cholesterol, and LDL cholesterol. In contrast, total cholesterol and LDL cholesterol were not significantly increased from baseline when a high calcium, high fat diet (25% of energy from SFA; 1971 mg calcium) was consumed. During the high calcium, high fat diet fecal calcium, bile acid, and fat excretion increased [32]. Therefore, it is likely that part of the attenuated lipid response observed with calcium consumption is due to increased fecal excretion of fatty acids and bile acids. 

Dairy fats include MCFA and fats not found in other foods [28], including butyric acid, phytanic acid, *cis*-palmitoleic acid and *trans*-palmitoleic acid [29]. Of these, butyric acid [33], *trans*-palmitoleic acid [34] and phytanic acid [35] have been shown to have antidiabetic properties. Phytanic acid, a product of ruminal degradation of chlorophyll, induces brown adipocyte differentiation, and induces the uncoupling protein in brown adipocytes, leading to increased thermogenesis [35]. This may decrease body fat and risk of CVD. In addition, both butyric acid and phytanic acid may act synergistically as peroxisome proliferator-activated receptors agonists, possibly acting synergistically to lower blood glucose levels [35], a factor that would increase insulin sensitivity. *Trans*-palmitoleate is inversely associated with triglycerides, fasting insulin, blood pressure and type 2 diabetes risk [36]. However, not all of dairy’s fatty acids have been shown to have beneficial effects. In an analysis of 788 matched pairs within the Physician’s Health Study, *cis*-palmitoleic acid was associated with an increased risk of heart failure [37].

#### 2.2.2. Replacing Dairy Fat with PUFA and Carbohydrate 

In a five-period, randomized controlled, crossover trial, the effect of consuming equal amounts of SFA from cheese (32% of energy from fat; 12.6% of energy from SFA) and butter (32% of energy from fat; 12.4% of energy from SFA) was compared to diets rich in MUFA (32% of energy from fat; 5.8% of energy from SFA; 19.6% of energy from MUFA), PUFA (32% of energy from fat; 5.8% of energy from SFA; 11.5% of energy from PUFA) and carbohydrate (58.9% of energy from carbohydrate, 25% of energy from fat; 5.8% of energy from SFA) [38]. After 4 weeks, total cholesterol and LDL cholesterol were significantly higher with the butter and cheese diets compared to the MUFA, PUFA and carbohydrate diets. Interestingly, LDL cholesterol was higher following consumption of the diet high in butter compared with the diet high in cheese, although the levels in both treatments were significantly higher than after the MUFA, PUFA or carbohydrate diets. This study suggests that consuming MUFA, PUFA or carbohydrate in place of SFA from dairy will reduce total and LDL cholesterol. In contrast, there was no difference in total, LDL or HDL cholesterol after 12 weeks when subjects with metabolic syndrome consumed either 80 g/d of full fat cheese or an isocaloric amount of carbohydrate [39]. The replacement of cheese with carbohydrate resulted in 7% of energy from fat (mainly SFA) being replaced with carbohydrate. Finally, in a 2-period randomized crossover study participants were provided with refined olive oil or butter equivalent to 4.5% of their energy requirement [40]. After 5 weeks, total cholesterol and LDL cholesterol were significantly higher with consumption of butter compared with refined olive oil. These studies suggest that replacing dairy fat with MUFA and PUFA is likely to have the most favorable effect on total and LDL cholesterol.

A recent analysis of data from the combined HPFS, the NHS and the Nurses’ Health Study II (NHS II) investigated replacing 5% of energy from dairy fat with different PUFA sources and carbohydrates (see Table 1). Replacing dairy fat with carbohydrates from refined starches and added sugar was not associated with increased or decreased risk of CHD, stroke or total CVD [41]. However, replacing dairy fat with carbohydrate from whole grains reduced the risk of CVD, CHD and stroke. Relative to other animal fats, dairy was found to have less impact on CVD. When 5% of energy from dairy fat was replaced with animal fat from non-dairy sources, risk of CHD increased by 6% (RR 1.06, 95% CI 1.02, 1.10). However, when 5% of energy from dairy fat was replaced with an isocaloric amount of PUFA, risk of CHD was reduced by 26% and CVD risk was reduced by 24% [41]. When the PUFA subtype was investigated, substitution of SFA with both *n*-*6* and α-linolenic acid (ALA) reduced CVD, CHD and stroke risk. However, replacing dairy fat with marine *n*-*3* PUFA only reduced the risk of CVD and CHD, but not stroke. These finding suggest that dairy fat should be replaced with PUFA or whole grains to reduce CVD risk.

## 3. MUFA

### 3.1. The Association between MUFA and CVD Risk 

Oleic acid represents approximately 92% of MUFA consumed in the U.S. [51]. While there are many dietary sources of oleic acid, olive oil and canola oil are commonly consumed plant sources containing a high percentage of oleic acid. MUFA are also found in animal products (meat and dairy), and since animal products are major sources of SFA as well, analyses can be confounded by the food sources. 

Clinical trial data evaluating the influence of MUFA on coronary artery disease (CAD) are lacking (as reviewed by Kris-Etherton and Fleming [15]), although there is evidence that higher consumption of MUFA improves risk factors for CVD. A meta-analysis of longer-term studies (>6 months) comparing consumption of high MUFA diets (>12% total calories) to low MUFA diets (≤12% calories) found that high MUFA diets were associated with lower fat mass, and lower systolic and diastolic blood pressure [52]. Similarly, Gillingham et al., reported that high MUFA diets (>15% total calories) were associated with increased HDL cholesterol, as well as decreased triglycerides and blood pressure [53]. Waist circumference was also decreased with high MUFA diets. With regards to body composition and weight, Liu et al., found reductions in android fat mass in a study of subjects at risk for metabolic syndrome [54]. The study was a randomized controlled, crossover feeding study comparing high MUFA intake to a high PUFA diet. Each of the five diets lasted four weeks, with a minimum washout of two weeks between the different diets. Compared to the high PUFA diet, the high MUFA treatments reduced android fat mass (especially in men) and were associated with a significant decrease in diastolic blood pressure. 

Epidemiological studies investigating the effect of MUFA on CVD have shown mixed results. Two meta-analyses of cohort studies found no significant association between MUFA and CHD events or death [8,55]. However, in a systematic review, Mente et al., showed a strong association between greater dietary MUFA intake and lower CHD risk (RR 0.80, 95% CI 0.67, 0.93), as well as an association between following the Mediterranean dietary pattern and lower CHD risk (RR 0.63, 95% CI 0.53, 0.72) [56]. The Prevención con Dieta Mediterránea (PREDIMED) trial also reported that diets higher in MUFA (either from 50 g/d extra-virgin olive oil or 30 g/d mixed nuts) compared with a lower fat diet reduced CVD events (about 30%), including myocardial infarction (MI) and stroke in high risk subjects [3]. 

MUFA are proposed to benefit cardiovascular health via several different mechanisms [57]. Among the suggested mechanisms are factors that alter the lipid/lipoprotein profile, such as inactivating sterol regulatory element binding protein (SREBP), a transcription factor that regulates cholesterol synthesis, and increasing expression of hepatic LDL receptor via stimulating acyl-CoA: cholesterol acyltransferase [58]. Kien et al., also found higher production of acylcarnitines in their female subjects on high oleic acid diets, suggesting a shift in fat catabolism [58]. Interestingly, MUFA have been shown to trigger greater diet-induced thermogenesis and greater fat oxidation rates compared to SFA [59]. 

### 3.2. Replacing SFA with MUFA

In a meta-analysis of randomized controlled trials, Mensink et al., showed that replacement of SFA with MUFA reduced total cholesterol, LDL and HDL cholesterol and triglycerides (see Table 2) [4]. However, as shown in Table 1 the effect of replacing SFA with MUFA on CVD, CVD mortality and all-cause mortality is less clear. Jakobsen et al., conducted a meta-analysis of 11 prospective cohort studies and found no effect of replacing SFA with MUFA on coronary events or death [42]. Similarly, a Cochrane review of randomized controlled trials showed no benefit of replacing SFA with MUFA [46]. Analyses from the PREDIMED cohort, and the NHS and HPFS showed that substitution of SFA with MUFA principally from plant sources reduced the risk of CVD [43,44]. Although replacement of specific SFA (12:0–18:0) with MUFA was not associated with any reduction in CHD risk in the NHS and HPFS cohorts [17], replacing SFA with MUFA increased the risk of IHD in the EPIC-Netherlands cohort [31]. Mixed results were also shown for the effect of this substitution on all-cause mortality. These inconsistent findings may be explained by fatty acid sources that not only vary among different populations but have also changed over time. An analysis from the NHS and HPFS showed that in the late 1980s the main source of MUFA was red meat, however by 2010 the main sources were vegetable oils and nuts [45].

## 4. PUFA

### 4.1. The Association between Total PUFA and CVD Risk 

The major types of PUFA include *n*-*3* and *n*-*6* PUFA. This section will present the evidence for the association between total PUFA intake (*n*-*3* + *n*-*6*) and CVD, or interventions that have simultaneously increased intake of *n*-*3* and *n*-*6* PUFA. A meta-analysis of randomized controlled trials that increased both *n*-*3* and *n*-*6* intake showed a reduced risk of non-fatal MI + CHD death (RR 0.78, 95% CI 0.65, 0.93) [60]. In the Kuopio Ischemic Heart Disease Risk Factor Study, intake of PUFA was inversely associated with fatal CHD in men, although no relationship existed with nonfatal CHD [61]. Similarly, in women involved in the NHS intake of PUFA in the highest quintile was associated with a 32% reduction in risk of CHD, relative to the lowest quintile [62]. A more recent follow-up of this cohort showed a similar magnitude of effect (25% risk reduction) [63]. In addition, a combined analysis of the NHS and HPFS cohorts showed a 20% reduction in CHD risk in the highest quintile of intake compared with the lowest [44]. However, some prospective cohort studies have shown that PUFA increase the risk of cardiovascular outcomes [31] or are not associated with risk [64]. Despite this, the totality of the evidence suggests that PUFA are protective against CVD. 

#### Replacing SFA with *n*-*3* + *n*-*6* PUFA

There is a large evidence base showing that replacement of SFA with mixed PUFA (*n*-*3* + *n*-*6*) reduces CVD risk (see Table 1 and Table 2). A systematic review and meta-analysis of randomized controlled trials showed that replacement of SFA with *cis*-PUFA reduced total cholesterol, LDL- and HDL-cholesterol, and triglycerides [4]. Mozaffarian et al., reported that for every 5% of energy from SFA exchanged for PUFA in short-term feeding trials, LDL cholesterol was reduced by 10 mg/dL, and the ratio of total cholesterol: HDL cholesterol decreased 0.16 [47]. These meta-analyses show that replacing SFA with PUFA improves the lipid profile, potentially reducing CVD risk.

Epidemiological studies and randomized controlled trials show that replacing SFA reduces the risk of cardiovascular events and death (see Table 1). In an analysis of prospective cohort studies, a 5% increase in energy intake from PUFA reduced the risk of a CHD event by 16%, however no association was observed with CHD death [55]. Skeaff and Miller also conducted a meta-analysis of randomized controlled trials that showed replacing SFA with PUFA reduced the risk of CHD events by 17%, although there was no effect on CHD death [55]. Similarly, a Cochrane review of randomized controlled trials showed that replacing SFA with PUFA reduced the risk of CVD events by 27% [46]. Furthermore, a subsequent meta-analysis of randomized controlled trials showed that for every 5% of energy from SFA replaced with an isocaloric amount of PUFA, the risk of CHD events was reduced by 10% [47]. Li et al.’s analysis of the NHS cohort (1980–2010) and the HPFS (1986–2010) indicated that replacing 5% of energy from SFA with an isoenergetic amount of PUFA was associated with a 25% lower CHD risk [44]. In another analysis from the NHS and HPFS cohorts, it was shown that replacing 1% of 12:0–18:0 SFA with PUFA reduced CHD risk by 8% [17]. Similarly, prospective cohort studies show that replacing SFA with PUFA reduces coronary events [42], CVD [43], total, CVD and coronary mortality [42,43,45].

### 4.2. The Association between n-6 PUFA and CVD Risk 

In a prospective cohort study of more than 91,000 women (NHS), there was an inverse association between *n*-6 PUFA and sudden cardiac death risk (SCD), independent of traditional CHD risk factors. When the highest quintile of PUFA intake was compared to the lowest quintile, the risk of SCD was reduced by 43% in those with a high intake (RR 0.57, 95% CI 0.41, 0.78) [65]. Findings from the Cardiovascular Health Study lend support to that study [66]. Wu et al., followed more than 2700 participants who were 65 or older and free of CVD at baseline, from 1992–2010 [66]. They found that higher plasma phospholipid linoleic acid concentrations were associated with lower mortality from CVD, especially mortality related to nonarrhythmic CHD (HR 0.51,95% CI 0.32, 0.82, *p* = 0.001). More importantly, they also found that when subjects were categorized based on both their plasma linoleic acid and *n*-*3* PUFA concentrations, those with the highest circulating levels of both had a 54% lower mortality risk (HR 0.46, 95% CI 0.30, 0.69) as compared to those with the lowest levels of both, demonstrating the importance of both fatty acids in the diet for cardiovascular health. 

#### Replacing SFA with *n*-*6* PUFA

Prospective cohort studies show concordant evidence that replacement of SFA with *n*-*6* PUFA reduces the risk of CVD events and mortality. A meta-analysis of prospective cohort studies showed that the risk of coronary events was reduced by 9% when 5% of energy from SFA was replaced with linoleic acid, and the risk of coronary death was reduced by 13% [48]. Most studies included in this meta-analysis not only adjusted for potential CHD risk factors, but also adjusted for energy obtained from other fat sources, including *trans*-fats. Similarly, in the NHS and HPFS, replacing 2% of energy from SFA with *n*-*6* PUFA was associated with a reduced risk of CVD mortality (−11%) and total mortality (−7%) [45]. This analysis of prospective cohort data likewise adds strong support to the evidence showing *n*-*6* PUFA reduce the risk of both CVD events and mortality.

The results of two recent secondary analyses investigating the effects of replacing SFA with linoleic acid, however, were not consistent with the previous studies, although these inconsistencies can be explained by the methodologies used. In the Sydney Diet Heart Study (1966–1973), individuals who had had an acute coronary event were randomized to either a high *n*-*6* PUFA, low SFA diet (PUFA 15% and SFA <10% of calories) or a usual intake control group. After approximately 3 years, participants randomized to the high PUFA diet had higher rates of all-cause, CHD and CVD death [67]. These divergent results are explained by the *trans*-fat present in the primary dietary source of linoleic acid (e.g., stick margarine). Ramsden et al., also published a re-analysis of the Minnesota Coronary Experiment (1968–1973) [68]. This trial was conducted in nursing homes and mental hospitals, and showed that replacement of SFA with *n*-*6* PUFA (corn oil and corn oil margarine; PUFA 13.2% and SFA 9.2% of calories) reduced total cholesterol, compared to a control high SFA low PUFA diet (SFA 18.5% and PUFA 4.7% of calories) [69]. Further, there was no difference in total mortality or cardiovascular mortality after a mean follow-up of one year. Recently, a re-analysis of this study was published, including only participants who were followed for longer than one year (approx. one quarter of the original sample), and it was found that those consuming the higher PUFA diet had a greater reduction in total cholesterol, but no mortality benefit. In both the intervention and control groups, greater cholesterol reduction was associated with a higher risk of mortality, but only in people older than 65 years [68]. It should be noted that the participants in this study are not representative of the general population because they were institutionalized and likely had multiple and complex morbidities. Also, this study had a large attrition rate and participants were only exposed to the intervention for a relatively short period of time (approx. 1 to 3 years). These two re-analysis studies received a lot of attention, however both have significant methodological flaws and therefore do not counter current recommendations to replace SFA with *n*-*6* PUFA. 

### 4.3. The Association between n-3 PUFA and CVD Risk 

#### 4.3.1. ALA (18:3 *n*-*3*)

Few clinical trials have evaluated the effect of ALA on CHD and CHD mortality. Despite this, there is growing evidence for a beneficial relationship between ALA and cardiovascular health. Currently, ALA intake in the U.S. is 1.8 g/d for men and 1.4 g/d for women, although increasing consumption to 2–3 g/d is recommended [70]. Foods high in ALA include walnuts (1 ounce provides 2.6 g ALA) and flax seeds (1 tablespoon provides 2.3 g ALA). 

In a meta-analysis of prospective and retrospective studies examining the association between ALA and CVD risk, Pan et al., demonstrated that each 1 g/d increase in dietary ALA was associated with a 10% lower risk of CHD death (RR 0.90, 95% CI 0.83, 0.99), although there was no association between CHD and blood or adipose tissue ALA levels [71]. An analysis of the NHS followed more than 76,000 women for 18 years, and after controlling for coronary risk factors and other dietary fatty acids (including long chain *n*-*3* PUFA) researchers found that higher ALA intake was related to lower rates of SCD (*p* = 0.02) [72]. For every 0.1% increase in energy from ALA, there was a 12% decrease in SCD (HR 0.88, 95% CI 0.80, 0.98). There was, however, no association between ALA intake and either fatal CHD or a non-fatal MI or total mortality [45,72]. In summary, prospective and retrospective studies report mixed findings for the association between ALA and CHD risk. Inconsistent results have also been shown in randomized controlled trials assessing the effect of ALA on CHD risk factors [15].

The effect of increased *n*-*3* consumption may depend on whether individuals are concurrently prescribed lipid lowering or anti-hypertensive medication. The Alpha Omega Trial assessed the effects of ALA on cardiovascular outcomes as compared to EPA + DHA, EPA + DHA + ALA or a placebo [73]. Patients (n = 4837) who had suffered a MI within the past ten years were assigned to one of three supplemented margarine treatments providing an average additional daily intake of 1.9 g ALA, or 226 mg EPA + 150 mg DHA, or a combination of all three. Participants were followed for 40 months, with an experimental end point of a fatal or non-fatal cardiovascular event, or a cardiac intervention. It is important to note that all patients also received lipid modifying, anti-hypertensive and anti-thrombotic treatments. During the 40-month follow-up, 13.9% of the participants suffered a major cardiovascular event. Although there was no significant effect of ALA (either alone or in combination with EPA + DHA), women in the two ALA treatments had a 27% reduction in major cardiovascular events that approached significance (HR 0.73, *p* = 0.07). Interestingly, a follow-up analysis of the Alpha Omega Trial evaluated how statin use (consistent use or consistent non-use) modified the impact of supplementation with ALA or EPA + DHA in patients with a previous MI [74]. In patients taking statins, there was no significant effect of *n*-*3* PUFA on cardiovascular events (adjusted HR 1.02, 95% CI 0.80, 1.32, P=0.88). In the group that did not use statins, the *n*-*3* supplemented groups were not different from controls when analyzed separately, but when all three intervention groups were combined (i.e., EPA + DHA; ALA; EPA + DHA + ALA), only 9% suffered a cardiovascular event as compared to 18% in the placebo group (adjusted HR 0.46, 95% CI 0.21, 1.01, *p* = 0.051). These results suggest statin treatment attenuates the *n*-*3* PUFA response [74], and that the addition of *n*-*3* PUFA may benefit patients not taking statins. 

#### 4.3.2. EPA (20:5 *n*-*3*) and DHA (22:6 *n*-*3*)

Current consumption of longer-chain marine *n*-*3* fatty acids in Americans is approximately 30 mg/d EPA + 60 mg/d DHA, a level that falls well short of current recommendations. The 2015–2020 Dietary Guidelines for Americans recommends 250 mg/d EPA + DHA [26]. This amount can be supplied by two servings (6 ounces or 168 g total) of fish/week, with one serving being from an oily fish such as salmon. It is important to consider the impact of replacing one food source for another. For example, if two 3-ounce servings of salmon replace two 3-ounce servings of high SFA meat, this will lower SFA intake by 9 g, increase PUFA by 8 g, and achieve the recommended EPA + DHA intake.

A meta-analysis of 20 randomized controlled trials showed that DHA + EPA protect against cardiovascular death (RR 0.86, 95% CI 0.75, 0.99, *p* = 0.03), although the analyses failed to find a significant decrease in composite CVD or coronary events (*p* = 0.24) [75]. A review of 21 clinical trials and randomized controlled trials, with a follow-up period of ≥6 months that mainly included individuals at high CVD risk, showed that consumption of marine-derived *n*-*3* PUFA (dietary or supplements) was associated with a 10% lower risk of any cardiovascular event (OR 0.90, 95% CI 0.85, 0.96, *p* = 0.001), a 9% lower risk of cardiac death (OR 0.91, 95% CI 0.83, 0.99, *p* = 0.03), and an 18% lower risk of fatal or non-fatal coronary events (OR 0.82, 95% CI 0.75, 0.90, *p* < 0.001) [76]. Although some meta-analyses have found no evidence that EPA and DHA reduces CVD risk [77,78,79], this is possibly because of differences in dosage, study duration or simultaneous statin usage [80]. 

Both clinical and epidemiological studies have found fish and fish oil reduce CAD death (~35%), CAD sudden death (~50%) and ischemic stroke (~30%) [15], although it is not clear what mechanisms are associated with these reductions. While the major cause of SCD is arrhythmia, recent reviews consistently find reductions in ventricular arrhythmias are not related to fish or fish oil consumption. 

#### 4.3.3. Replacement of SFA with *n*-*3* PUFA

Few studies have evaluated the effect of replacing SFA with *n*-*3* PUFA. Principally because *n*-*3* PUFA are found in relatively small amounts in dietary sources, and thus make up a small proportion of dietary fat intake, they are not a viable substitute for SFA. However, in the Lyon Diet Heart Study, a Mediterranean diet that included ALA and oleic acid in place of SFA, the rate of cardiovascular death and overall mortality was reduced after approximately 2 years of follow-up in individuals that had experienced a MI [81]. An analysis from the NHS and HPFS showed that replacing 0.3% of energy from SFA with *n*-*3* PUFA (total) was associated with a reduction in all-cause mortality by 5% but not for CVD mortality [45] (see Table 1).

## 5. Carbohydrate

### 5.1. Carbohydrate and CVD Risk

A clear relationship between intake of carbohydrates and CVD has not been observed [44,82,83]. This is because refined carbohydrates and whole grains have differing cardiometabolic effects, characteristics that are acknowledged in dietary recommendations. The Dietary Guidelines for American’s (2015–2020) recommend that 6 oz-eq/d of grains is consumed, and less than half should be refined grains. Another way of classifying carbohydrate-containing foods is in terms of the Glycemic Index (GI). GI is a ranking of carbohydrates according to how they affect blood glucose levels, relative to the standard reference carbohydrate, glucose. Foods with a lower GI are more slowly digested and absorbed, and therefore the postprandial increase in blood glucose levels is attenuated. Typically, foods that contain dietary components such as soluble fiber, plant cell walls and polyphenols have lower GIs. However, once a food is cooked (e.g., white potatoes), refined (e.g., white flour) or processed (e.g., sugars added), its monosaccharides, disaccharides and starches are more efficiently absorbed due to disruption and removal of molecules that decrease digestion rate. Glycemic Load (GL) is a measure that considers both the food’s GI and the amount of food (i.e., amount of carbohydrate) ingested. Studies discussed in this section either conducted analyses by looking at intake of whole grains vs. refined grains or low GI vs. high GI. 

Data from randomized controlled trials provide weak evidence that low GI diets improve total cholesterol compared to higher GI diets. A Cochrane review showed no benefit of low GI diets on LDL or HDL cholesterol or triglycerides [84]. More recently, Sacks et al., performed a 4-week randomized, cross-over controlled feeding trial (OmniCarb) comparing high GI and low GIs diet containing different amounts of carbohydrate [85]. The four diets were high carbohydrate (58% of dietary energy), with either a high GI (GI 66, GL 172) or low GI (GI 41, GL 104), or low carbohydrate (40% of dietary energy), with either a high GI (GI 65, GL 112) or low GI (GI 40, GL 64). In the low carbohydrate diets, protein and MUFA replaced carbohydrate. SFA remained at 6–7% of energy in all diets. As compared to the high carbohydrate/high GI diet, the high carbohydrate/low GI diet groups exhibited 20% lower insulin sensitivity (*p* = 0.002) and a 6% increase in LDL cholesterol (*p* ≤ 0.001). There were no significant differences in HDL cholesterol, triglycerides or blood pressure when comparing the low and high GI groups of the high carbohydrate treatment. Compared to the low carbohydrate/high GI diet, there was a 5% decrease in triglycerides with the low carbohydrate/low GI diet (*p* = 0.02). This study showed that a DASH style diet containing low GI foods does not improve CVD risk factors (and unexpectedly increased LDL cholesterol). 

Prospective cohort studies have shown mixed results for the association between high and low GI diets and CHD or stroke risk. A meta-analysis of 15 prospective cohort studies showed that higher dietary GI (RR 1.13; 95% CI 1.04, 1.22) and GL (RR 1.28; 95% CI 1.14, 1.42) were associated with an increased risk of CHD [86]. In addition, higher dietary GL was associated with greater stroke risk (RR 1.19; 95% CI 1.00, 1.43). A similar meta-analysis that included prospective cohort studies showed that those in the highest GI quantile versus the lowest quantile did not have an increased risk of CHD (RR 1.11; 95% CI 0.99, 1.24, *p* = 0.09), however, CHD risk was higher in individuals in the highest GL quantile compared with the lowest (RR 1.27; 95% CI 1.09, 1.49) [87]. Both of these meta-analyses reported a gender effect such that high GI and GL diets were associated with increased risk of CHD in women but not men. 

The mixed results that have been observed in prospective cohort studies and randomized controlled trials with regard to the cardiometabolic effect of GI and GL may be explained by the variability in individual responses to GI values assigned to food products. Matthan et al., studied the glycemic response to a single food item in 63 healthy volunteers, and found substantial variability in their responses (20% within an individual and 25% between individuals) [88]. The authors suggest that the high degree of variability in glycemic response means that GI is unlikely to be a good method to inform food choices. This high degree of variability has also been shown in individuals with type 2 diabetes [89]. 

Carbohydrates may also be classified in terms of whole grains and refined carbohydrates. A meta-analysis of 18 prospective studies showed that higher whole grain intake was associated with a reduced risk of CHD (RR 0.79; 95% 0.74, 0.83) [90]. In a more recent analysis, Benisi-Kohansal et al., showed that greater consumption of whole grains was associated with an 11% reduction in all-cause mortality (RR 0.89; 95% 0.84, 0.94), and a 16% reduction in CVD mortality (RR 0.84; 95% CI 0.76, 0.93) [91]. Similarly, Aune et al., showed in a meta-analysis of prospective cohort studies that there was an inverse association between whole grain intake and CHD, CVD, and mortality from CHD, stroke and all-causes [92]. In this meta-analysis, the effect of refined grains was also examined. Intake of refined grains was not associated with CHD (RR 1.13; 95% CI 0.90, 1.42), stroke (RR 0.91; 95% CI 0.81, 1.02) or CVD (RR 0.98; 95% CI 0.90, 1.06). There is also consistent evidence showing the greater intake of added sugar is associated with higher risk of CVD. An analysis from the NHS showed that compared with consumption of less than 5% of calories from added sugar, intakes of 10–24%, and ≥25% of total calories increased the risk of CVD mortality by 30% and 275%, respectively, after adjustment for traditional CVD risk factors [93]. Further, sugar-sweetened beverage consumption has been positively associated with hypertension and CHD [94,95,96]. A meta-analysis of randomized controlled trials found that higher intake of sugar significantly increased triglycerides, total cholesterol, LDL cholesterol and HDL cholesterol [97]. In summary, the totality of the evidence shows whole grains are protective against CVD, a finding that is in contrast to refined grains and added sugar that have no effect or increase CVD risk, respectively.

High GI/GL foods and added sugars may increase CVD risk due to the rapid glucose digestion, resulting in high blood glucose levels that in turn trigger pancreatic insulin release while inhibiting glucagon release. The resulting high insulin: glucagon ratio is thought to trigger hypoglycemia, increase lipogenesis and decrease glucose oxidation rate [98]. Insulin activates sterol regulatory element-binding protein-1c (SREBP-1c). SREBP-1c is a transcription factor regulating fatty acid and triglyceride synthesis [99]. High postprandial glucose levels could be a risk factor for CVD, an association that has been identified at the population level [100]. Specifically, researchers performed a meta-regression with more than 95,000 individuals who were followed for an average of 12.4 years. They found that even after removing individuals whose resting or 2-h glucose levels classified them as having impaired glucose metabolism, higher 2-h levels of blood glucose were significantly associated with a greater risk of a cardiovascular event (*p* = 0.00064). In a study of overweight individuals, consumption of sucrose- sweetened beverages was associated with significant increases in inflammatory markers such as haptoglobin (13%) and transferrin (5%) [101]. The role of inflammation in the pathogenesis of atherosclerosis and CVD has been well established [102]. 

### 5.2. Replacing SFA with Carbohydrates

As previously stated, the association between SFA and CVD has recently been questioned by some largely on the basis of studies that have not considered what is being consumed instead of SFA. In many cases, SFA are replaced with carbohydrates predominately from refined starches and added sugars [44], a factor that may explain the null association recently observed between SFA and CVD. 

A meta-analysis of 60 randomized controlled trials showed that isocaloric replacement of SFA with carbohydrates did not result in a change in the ratio of total cholesterol: HDL cholesterol (i.e., it remained unfavorable) [18] (see Table 2). Additionally, this meta-analysis found replacing SFA with carbohydrates increased fasting triacylglycerol concentrations. Micha and Mozaffarian also found no overall benefit for replacing SFA with carbohydrates; relative to carbohydrates, SFA raise total cholesterol, LDL cholesterol and HDL cholesterol while lowering triglycerides [19]. 

Mixed results have been observed when total carbohydrates replace SFA (see Table 1). In a pooled analysis of 11 cohort studies from America and Europe replacing 5% of energy from SFA with carbohydrates increased the risk of coronary events, but no change in risk of coronary death was observed [42]. Similarly, in the EPIC-Netherlands cohort replacing 5% of energy from SFA with carbohydrate was associated with an increased risk of IHD [31]. In contrast, replacing 5% of calories from SFA with carbohydrate was not associated with a reduction in risk of MI in a Danish cohort [49]. A Cochrane review of randomized controlled trials also showed no change in risk of CVD events when SFA were replaced with carbohydrates [46]. In the PREDIMED cohort, Guasch-Ferre et al., showed no effect of replacing 5% of energy from SFA with carbohydrates on CVD and all-cause death [43]. 

In the NHS and HPFS cohorts, replacing 5% of energy from SFA with equivalent energy from whole grains was associated with a decreased risk of CHD while replacing 5% of energy from SFA with an isocaloric amount of carbohydrates from refined starches/added sugars did not change CHD risk (*p* > 0.10) [44]. Similarly, replacing 1% of energy from 12:0–18:0 SFA with whole grains was associated with a 6% reduction in CHD risk in another analysis from the NHS and HPFS cohorts [17]. Interestingly, an analysis from the EPIC-Netherlands cohort showed that replacing SFA with medium (GI 53–56) or high GI carbohydrates (GI > 56) was associated with a 35% and 27% greater risk of IHD, respectively. Replacing SFA with low GI carbohydrates (GI < 53) was not associated with a change in IHD risk [31]. Similarly, Jakobsen et al., found no significant change in MI risk when 5% of energy from SFA was replaced by low GI (median GI 82) or medium GI (median GI 88) carbohydrates [49]. However, MI risk was increased by 33% when 5% of energy from SFA was replaced with high GI carbohydrates (median GI 93). In summary, replacing SFA with whole grains confers a significant reduction in CHD risk, whereas refined grains and low GI carbohydrates do not change risk. Conversely, high GI carbohydrates increase the risk of MI and IHD. 

## 6. Protein

### 6.1. Protein and CVD Risk

The 2010 Dietary Guidelines for Americans first recommended a shift towards a more plant-based diet that included protein sources such as legumes, nuts and seeds and whole grains. The 2015 Dietary Guidelines Advisory Committee further suggested that calories from added sugars be partially replaced by consuming a wider variety of plant proteins [103].

To investigate the association between dietary protein and IHD, Hu et al., followed more than 80,000 healthy women aged 34–59 years in the NHS cohort from 1980 to 1994 [16]. After adjusting the data for age, smoking status, total energy intake and the percent of energy intake from fats (SFA, MUFA, PUFA and *trans*), they found that relative to those consuming the lowest amounts of protein, women who consumed the highest amounts of total protein (plant + animal) had the lowest risk of IHD (RR 0.74, 95% CI 0.59, 0.95, *p* < 0.05). Both animal and vegetable proteins contributed to the lower disease risk. However, it is interesting to note that among the participants, the largest dietary contributors to plant protein were dark bread (8%), white bread (7%), and ready-to-eat cereals (5%), foods often associated with higher carbohydrate loads. 

Similar findings associating higher protein intake with lower CVD risk were reported in a cross-sectional study of 1898 female twins aged 18–75 years [104]. After adjusting for confounding variables, they found that greater total dietary protein intake (plant + animal, lowest quintile 12.8 ± 1.1% of energy intake, highest quintile 19.9 ± 1.5% of energy intake) was associated with a 3 mm Hg decrease in central systolic blood pressure (*p* < 0.01), a 2.4 mm Hg decrease in mean arterial pressure (*p* < 0.01), and a 1.9 mm Hg decrease in diastolic blood pressure (*p* < 0.01). Although small, a 2 mm Hg reduction in blood pressure was associated with a 4% decrease in total mortality in the INTERSALT study [105]. 

Higher protein consumption has not been associated with decreased CVD risk in all studies. A secondary analysis was performed in the PREDIMED cohort, a parallel-group, randomized controlled trial conducted on 7216 adults who were at risk of developing CVD [106]. Analytical regression models were adjusted for sex, BMI, smoking status, family history of disease, and either the percentage of total dietary energy from fats or the percentage of energy from carbohydrates. When models were adjusted for the percentage of energy from fat, those in the highest protein intake quintile had a 66% greater risk of death (combined CVD and cancer, *p* < 0.001) relative to the middle quintile. When models were adjusted for percentage of energy from carbohydrate, those in the highest protein intake quintile had a 59% increased risk of death (combined CVD and cancer, *p* < 0.001) relative to the middle quintile. Despite this combined effect, dietary protein was not a factor in separate measures of cardiovascular events, CVD deaths or cancer deaths. 

While total protein may not consistently predict CVD risk, several studies have demonstrated that plant and animal proteins differ in their association with CHD. Kelemen et al., followed more than 29,000 postmenopausal women for 15 years [107]. Their analysis showed a 30% reduction in CHD among participants when plant proteins were substituted isoenergetically for carbohydrates, or when plant proteins were isoenergetically substituted for animal proteins (*p* = 0.02). In a prospective cohort study, Preis et al., found a similar inverse association between plant protein and risk of fatal IHD in middle-aged men [108]. Although Preis et al., found no association between the percentage of energy from total protein, animal protein or vegetable protein and the risk of IHD, they did find a significant inverse relationship between consuming greater amounts of vegetable protein and a lower risk of fatal IHD (RR 0.66, 95% CI 0.49, 0.88, *p* < 0.005) [108]. Additionally, in men without hypertension and in those consuming a low GI diet, total protein and animal protein consumption were related to a greater risk of IHD. A related study on the same cohort did not, however, find any significant association between total protein, animal protein or vegetable protein and risk of stroke [109].

A recent analysis from the NHS and HPFS showed protein from plant sources was more protective against cardiovascular mortality than protein from animal sources [110]. In individuals who had at least one unhealthy lifestyle factor (e.g., smoking, high alcohol intakes, overweight, obesity, physical inactivity), consuming animal protein was associated with higher mortality, especially cardiovascular mortality (HR 1.08 per 10% energy increment, 95% CI 1.01, 1.16, *p* = 0.04 for trend). However, consuming plant protein was associated with lower mortality risk in this group (HR 0.90 per 3% energy increment; 95% CI 0.86, 0.95; *p* < 0.001 for trend). 

Unlike the Kelemen et al. [107], Preis et al. [108] and Song et al. [110] studies that found a protective role of plant protein against CVD, the PREDIMED analysis [106] did not find a significant association between plant protein intake and CVD, although their analysis indicated that animal proteins were detrimental for cardiovascular health. When Hernández-Alonso et al., adjusted the models for percentage of energy from carbohydrates or fats, consuming a higher percentage of energy from animal protein was positively associated with higher risk of fatal and non-fatal CVD events. However, percentage of energy from vegetable protein was not significant in predicting fatal or non-fatal CVD events in either of these adjusted models. Unlike previous research that indicated higher protein is associated with increased weight loss, at least in the short term [111], the PREDIMED analysis [106] found that both BMI and bodyweight were positively associated with the percentage of energy from total protein intake, protein derived from animal sources, and the ratio of animal-to-vegetable protein. Vegetable protein intake, however, was not associated with increased BMI or bodyweight. The influence of animal protein on BMI and body weight may explain, in part, the positive association between animal protein and CVD risk in this study.

While some studies have shown that animal protein, in general, adversely affects cardiovascular health, a number of studies have indicated that we should also consider the type of animal protein and whether or not the meat is processed when considering CVD risk. The National Institutes of Health—American Association of Retired Persons Diet and Health Study followed ~500,000 men and women who were 50 to 71 years old for 10 years [112]. Compared to the lowest intake quintile, people in the highest quintile of red meat consumption had an elevated CVD risk (men: HR 1.27, 95% CI 1.20, 1.35; women: HR 1.50, 95% CI 1.37, 1.65). High processed meat intakes also increased CVD risk (men: HR 1.09, 95% CI 1.03, 1.15; women: HR 1.38, 95% CI 1.26, 1.51). Pan et al., also reported an increased CVD mortality risk for both unprocessed red meat (HR 1.18, 95% CI 1.13, 1.23) and processed red meat (HR 1.21, 95% CI 1.13, 1.31) [113]. In a related study, Song et al., examined the impact of replacing 3% of the energy from animal proteins with an isocaloric amount of plant-based proteins [110]. Besides indicating the protective nature of consuming plant-based proteins, they also reported very different mortality risks when plant proteins replaced either the more harmful processed red meats (HR 0.66, 95% CI 0.59, 0.75) or less harmful unprocessed red meat (HR 0.88, 95% CI 0.84, 0.92).

Not all studies, however, found both unprocessed meat and processed meat to be significant predictors of CVD. The EPIC study found that processed meats, but not red meat or white meat intake, was associated with higher CVD mortality (HR processed meats 1.18, 95% CI 1.11, 1.25) [114]. Similarly, Kaluza et al., examined a cohort of more than 37,000 Swedish men and found processed meat, but not unprocessed meat, was positively associated with risk of heart failure [115]. Specifically, men consuming ≥75 g/d processed meat, as compared to those consuming <25 g/d, had a 28% greater risk of heart failure (HR 1.28, 95% CI 1.10, 1.48, *p* = 0.01), and a much higher risk of heart failure mortality (HR 2.43, 95% CI 1.52, 3.88, *p* < 0.001). Consuming unprocessed meat was not associated with heart failure incidence or heart failure mortality.

Similar to Sinha et al. [112], Pan et al. [113] and Song et al. [110], the NHS [116] also found higher intakes of red meat (both including and excluding processed meat), were associated with increased CHD risk. Interestingly, this study also found higher intakes of poultry, fish and nuts were associated with a lower CHD risk. Compared to the quintile with the lowest intake of poultry, a multivariable model indicated the risk of developing CHD for those in the highest quintile of poultry consumption was reduced by 8% (*p* = 0.02). Similarly, for those in the highest quintile of fish consumption and nut consumption, the risk of CHD was reduced by 19% (*p* < 0.001) and 32% (*p* < 0.001), respectively. 

Dietary protein sources comprise a heterogenous category of foods that contain a variety of non-protein compounds that impact cardiometabolic risk factors. Therefore, the different cardiometabolic effects observed for plant and animal protein sources are likely to be explained by the nonprotein components rather than protein per se [117]. Generally, plant-based protein sources are low in SFA, and also contain micronutrients (e.g., magnesium, potassium, carotenoids, vitamin C, B vitamins), phytosterols and polyphenols. Higher magnesium intakes are associated with lower risk of CVD [118], as are greater potassium intakes (relative to lower sodium intakes [119]). Polyphenols and phytosterols are also known to benefit the cardiovascular system [120,121,122]. In addition, the different amino acid profiles of plant and animal proteins may also contribute to the differing vascular effects. For example, plant proteins tend to be lower in the sulfur-containing amino acids like methionine, as well as tryptophan, threonine, lysine and leucine [123]. High levels of lysine and methionine, typically higher in animal proteins, have been shown to induce hypercholesterolemia in rabbits [124]. Leucine, also more common in animal tissues, has been shown to inhibit nitric oxide synthesis in the vascular endothelium, and may also promote insulin resistance [125]. Jennings et al. modeled the relationship between seven individual amino acids and blood pressure or indices of arterial stiffness in a cohort of women [104]. Higher intake of all seven amino acids (arginine, cysteine, glutamic acid, glycine, histidine, leucine, and tyrosine) was associated with lower central and peripheral blood pressure. In further analyses, the effect of these amino acids when derived from plant and animal sources on blood pressure and arterial stiffness was investigated. When comparing the lowest and highest intake quintiles, individual amino acids derived from plant sources were associated with significantly lower central and peripheral blood pressure and less arterial stiffness in many cases. In contrast, amino acids derived from animal sources were only significantly correlated with decreased blood pressure measures in a few comparisons. Therefore, it is likely that relative amounts and types of amino acids found in plant protein sources have a more favorable effect on cardiovascular risk factors.

Studies have shown that red meat, especially processed meats, are associated with greater CVD risk. However, other animal protein sources either have a neutral or protective effect. There are many suggested mechanisms that explain why red and processed meat may increase the risk of cardiometabolic disease; these have been comprehensively reviewed by Kim et al. [126] and Wolk [127]. In brief, in addition to being high in SFA, red and processed meats provide heme-iron, and higher intake levels have been associated with greater CVD risk [128]. Processed meat is also extremely high in sodium, and contains approximately 400% more sodium than unprocessed meat [129]. Sodium is a well-established risk factor for CVD [130]. In addition, nitrates and nitrites are present in processed meat and have also been shown to adversely affect cardiometabolic health [131]. Furthermore, advanced glycation end products formed during the cooking of red and processed meat may also contribute to the observed effect on CVD by increasing inflammation [132]. Finally, red meat provides L-carnitine and phosphatidylcholine that are metabolized to trimethylamine N-oxide, a compound associated with increased risk of CVD [133].

### 6.2. Replacement of SFA with Protein

Few studies have investigated the effect of replacing SFA with protein. In a Cochrane review of randomized controlled trials, replacing SFA with protein did not reduce the risk of CVD events [46]. In a prospective cohort study conducted in Sweden, it was found that replacing 5% of energy from SFA with protein reduced stroke risk [50]. Conversely, in the EPIC-Netherlands cohort, replacing 5% of energy from SFA with protein increased the risk of IHD by 29%. When protein type was examined, it was found that only substitution with animal protein was associated with increased risk of IHD [31]. Replacing 5% of energy from SFA with vegetable protein was not associated with a change in risk. However, in an analysis from the NHS and HPFS, replacing 1% of energy from 12:0–18:0 SFA with plant protein reduced the risk of CHD by 7% [17].

## 7. Conclusions

Although Americans have reduced their consumption of SFA since the early 1970s [134], current intake exceeds dietary recommendations (2015–2020 Dietary Guidelines for Americans [26]). Prospective studies and randomized controlled trials provide strong evidence that replacing dietary SFA with unsaturated fatty acids, both MUFA and PUFA, and carbohydrates from fiber rich whole grains benefit cardiovascular health. Thus, as healthcare providers recommend that patients reduce dietary SFA, it is imperative that they suggest isocaloric replacements for SFA calories that will have the greatest impact on improving patient health. There is a strong evidence base for CVD risk reductions when SFA are replaced by PUFA (with sufficient *n*-*3* PUFA). Although the benefits of MUFA have not been as strongly supported as benefits from PUFA, there is growing evidence that replacing SFA with MUFA from plant sources decreases CVD risk. Other substitutions for dietary SFA to decrease CVD risk include carbohydrates from whole grains. Refined carbohydrates should not be substituted for SFA since they do not confer any benefit.

Further research is needed about the cardiovascular benefits of replacing SFA with protein. Of note is that the type of protein substituted may affect CVD risk. For instance, increasing plant proteins has been shown to be more beneficial than animal proteins, although fish and seafood (classified as an animal protein) are recommended at least twice a week because of their health benefits. Despite popular press suggestions that dairy fat is beneficial, this appears to be relative to red meat. Replacing dairy fat with PUFA decreases CVD risk. In addition, cheese and fermented dairy products are less hypercholesterolemic than butter, although PUFA and MUFA lower total and LDL cholesterol compared with cheese and butter. In summary, there are many options for reducing SFA in the diet and replacing these calories isocalorically with macronutrients, including unsaturated fats (PUFA and MUFA), carbohydrate from whole grains, and protein with emphasis on plant sources will reduce CVD risk. Further research will help identify the nutrient(s) replacement(s) for SFA to maximize CVD risk reduction.

## Figures and Tables

**Table 1:** The effect of replacing SFA with other dietary macronutrients on cardiovascular outcomes.

Study	Design	*n*	Mean Follow-Up Time (Years)	Outcome	Substitution	Result	Effect Size (95% CI)	Covariates Included in Analyses
**Substitution of Saturated Fat for MUFA**								
Jakobsen 2009 [42]	Pooled analysis of prospective cohort studies	11 studies (*n* = 344,696)	Range 4 to 10	Coronary events	5% of energy from SFA → MUFA	↔	HR 1.19 (1.00–1.42)	Age; BMI; year survey completed; percentage of energy from MUFA, PUFA, *trans*-fat, protein and carbohydrates; energy intake; smoking; physical activity; education; alcohol intake; fiber intake; cholesterol intake; hypertension
				Coronary deaths		↔	HR 1.01 (0.73–1.41)	
Guasch-Ferré 2015 [43] [PREDIMED]	Prospective cohort	7038	6	CVD	5% of energy from SFA → MUFA	↓	HR 0.63 (0.43–0.94)	Age; sex; BMI; intake of subtypes of fat, protein, and carbohydrates; energy intake; smoking; physical activity; education; alcohol intake; fiber intake; cholesterol intake; hypertension; intervention group; diabetes; hyper-cholesterolemia; family history of CHD; antihypertensive medication; oral antidiabetic agents; lipid lowering drugs
				All-cause death		↔	HR 0.91 (0.65–1.26)	
Li 2015 [44] [NHS; HPFS]	Prospective cohort	127,536	Range 24–30	CHD	5% of energy from SFA → MUFA	↓	HR 0.85 (0.74–0.97)	BMI, percentage of energy from protein; energy intake; smoking; physical activity; alcohol intake; cholesterol intake; hypertension at baseline; hypercholesterolemia at baseline; family history of myocardial infarction and diabetes; use of vitamins and aspirin
Praagman 2016 [31] [EPIC-Netherlands]	Prospective cohort	35,597	12	IHD	5% of energy from SFA → *cis*-MUFA	↑	HR 1.30 (1.02–1.65)	Age, sex, BMI, waist circumference; intake of carbohydrate, *cis*-MUFA, PUFA, *trans*-fat, animal protein and vegetable (per 5% of energy); energy intake (excluding alcohol); smoking, physical activity; education; alcohol intake; fiber intake (energy adjusted); cholesterol intake (energy adjusted); vitamin c (energy adjusted)
Wang 2016 [45] [NHS; HPFS]	Prospective cohort	126,233	NHS ≤ 32; HPFS ≤ 26	CVD mortality	5% of energy from SFA → MUFA	↔	HR 0.96 (0.84–1.09)	Age; BMI, percentage of energy intake from protein, remaining fatty acids (saturated fat, PUFA, MUFA, *trans*-fat, ω-6 PUFAs, ω-3 PUFAs, linoleic acid, arachidonic acid, α-linolenic acid, and marine ω-3 fats); energy intake; smoking; physical activity; alcohol intake; cholesterol intake; family history of myocardial infarction, diabetes, cancer, hypertension, hyper-cholesterolemia; multivitamin use; vitamin E supplement; aspirin use; white race; marital status; menopausal status and hormone use in women
				Total mortality		↓	HR 0.87 (0.82–0.93)	
Zong 2016 [17] [NHS; HPFS]	Prospective cohort	115,782	NHS 25.8; HPFS 21.2	CHD	1% of energy from 12:0–18:0 SFA → MUFA	↔	HR 0.95 (0.90, 1.01)	Age; BMI; ethnicity; total energy; energy from *trans*-fat; energy from carbohydrates of non-whole grain sources; energy from non-plant sources; smoking status; physical activity; alcohol intake; family history of MI; menopausal status; postmenopausal hormone use; aspirin use; multivitamin use; baseline hypertension; baseline hypercholesterolemia; PUFA intake; whole grains intake; plant proteins intake; intake of other SFA
Hooper 2015 [46] Cochrane review	Meta-analysis of randomized controlled trials	15 studies (*n* > 59,000)	>2	CVD events	SFA → MUFA	↔	RR 1.00 (0.53–1.89)	Aggregate meta-analysis—no overall adjustment
**Substitution of saturated fat for PUFA**								
Mozaffarian 2010 [47]	Meta-analysis of randomized controlled trials	8 studies (*n* = 13,614)	Median of all trials 4.25	CHD	5% of energy from SFA → total PUFA	↓	RR 0.90 (0.83–0.97)	Aggregate meta-analysis—no overall adjustment
Jakobsen 2009 [42]	Pooled analysis of prospective cohort studies	11 studies (*n* = 344,696)	Range 4 to 10	Coronary events	5% of energy from SFA → total PUFA	↓	HR 0.87 (0.77–0.97)	Age; BMI; year survey completed; percentage of energy from MUFA, PUFA, *trans*-fat, protein and carbohydrates; energy intake; smoking; physical activity; education; alcohol intake; fiber intake; cholesterol intake; hypertension
				Coronary deaths		↓	HR 0.74 (0.61–0.89)	
Farvid 2014 [48]	Meta-analysis of prospective cohort studies	13 studies (*n* = 310,602)	Range 5.3 to 30	Coronary events	5% of energy from SFA → linoleic acid	↓	RR 0.91 (0.87–0.96)	Aggregate meta-analysis—analyses in the individuals studies adjusted but no overall adjustment
				Coronary deaths		↓	RR 0.87 (0.82–0.94)	
Li 2015 [44] [NHS; HPFS]	Prospective cohort	127,536	Range 24–30	CHD	5% of energy from SFA → total PUFA	↓	HR 0.75 (0.67–0.84)	BMI, percentage of energy from protein; energy intake; smoking; physical activity; alcohol intake; cholesterol intake; hypertension at baseline; hypercholesterolemia at baseline; family history of myocardial infarction and diabetes; use of vitamins and aspirin
Guasch-Ferré 2015 [43] [PREDIMED]	Prospective cohort	7038	6	CVD	5% of energy from SFA → PUFA	↓	HR 0.67 (0.45–0.98)	Age; sex; BMI; intake of subtypes of fat, protein, and carbohydrates; energy intake; smoking; physical activity; education; alcohol intake; fiber intake; cholesterol intake; hypertension; intervention group; diabetes; hyper-cholesterolemia; family history of CHD; antihypertensive medication; oral antidiabetic agents; lipid lowering drugs
				All-cause mortality		↓	HR 0.61 (0.39–0.97)	
Chen 2016 [41] [NHS; NHS II; HPFS]	Prospective cohort	134,327	NHS ≤ 32; NHS II ≤; HPFS ≤ 24	CVD	5% of energy from dairy fat → total PUFA	↓	HR 0.76 (0.71–0.81)	Age, BMI, intake of protein; energy intake; smoking; physical activity; intake of fruit, vegetables, coffee; alcohol intake; baseline hypertension; baseline hyper-cholesterolemia; race; menopausal status and menopausal hormone use (NHS and NHS II); oral contraceptive use (NHS II only)
				CHD		↓	HR 0.74 (0.68–0.81)	
				Stroke		↓	HR 0.78 (0.70–0.88)	
				CVD	5% of energy from dairy fat → *n*-*6* PUFA	↓	HR 0.75 (0.70–0.81)	
				CHD		↓	HR 0.75 (0.69–0.82)	
				Stroke		↓	HR 0.76 (0.68–0.86)	
				CVD	0.3% of energy from dairy fat → α-linolenic acid	↓	HR 0.86 (0.82–0.90)	
				CHD		↓	HR 0.83 (0.78–0.88)	
				Stroke		↓	HR 0.89 (0.83–0.96)	
				CVD	0.3% of energy from dairy fat → marine *n*-*3*	↓	HR 0.89 (0.84–0.94)	
				CHD		↓	HR 0.87 (0.81–0.93)	
				Stroke		↔	HR 0.92 (0.84–1.01)	
Praagman 2016 [31] [EPIC-Netherlands]	Prospective cohort	35,597	12	IHD	5% of energy from SFA → PUFA	↑	HR 1.35 (1.14–1.61)	Age, sex, BMI, waist circumference; intake of carbohydrate, *cis*-MUFA, PUFA, *trans*-fat, animal protein and vegetable (per 5% of energy); energy intake (excluding alcohol); smoking, physical activity; education; alcohol intake; fiber intake (energy adjusted); cholesterol intake (energy adjusted); vitamin c (energy adjusted)
Wang 2016 [45] [NHS; HPFS]	Prospective cohort	126,233	NHS ≤ 32; HPFS ≤ 26	CVD mortality	5% of energy from SFA → total PUFA	↓	HR 0.72 (0.65–0.80)	Age; BMI, percentage of energy intake from protein, remaining fatty acids (saturated fat, PUFA, MUFA, *trans*-fat, ω-6 PUFAs, ω-3 PUFAs, linoleic acid, arachidonic acid, α-linolenic acid, and marine ω-3 fats); energy intake; smoking; physical activity; alcohol intake; cholesterol intake; family history of myocardial infarction, diabetes, cancer, hypertension, hyper-cholesterolemia; multivitamin use; vitamin E supplement; aspirin use; white race; marital status; menopausal status and hormone use in women
				Total mortality		↓	HR 0.73 (0.70–0.77)	
				CVD mortality	2% of energy from SFA → *n*-*6* PUFA	↓	HR 0.89 (0.85–0.94)	
				Total mortality		↓	HR 0.93 (0.91–0.96)	
				CVD mortality	0.3% of energy from SFA → *n*-*3* PUFA	↔	HR 1.01 (0.97–1.05)	
				Total mortality		↓	HR 0.95 (0.93-0.96)	
Zong 2016 [17] [NHS; HPFS]	Prospective cohort	115,782	NHS 25.8; HPFS 21.2	CHD	1% of energy from 12:0–18:0 SFA → PUFA	↓	HR 0.92 (0.89, 0.96)	Age; BMI; ethnicity; total energy; energy from *trans*-fat; energy from carbohydrates of non-whole grain sources; energy from non-plant sources; smoking status; physical activity; alcohol intake; family history of MI; menopausal status; postmenopausal hormone use; aspirin use; multivitamin use; baseline hypertension; baseline hypercholesterolemia; MUFA intake; whole grain intake; plant protein intake; intake of other SFA
Hooper 2015 [46] Cochrane review	Meta-analysis of randomized controlled trials	15 studies (*n* > 59,000)	>2	CVD events	SFA → PUFA	↓	RR 0.73 (0.58–0.92)	Aggregate meta-analysis—no overall adjustment
**Substitution of Saturated Fat for Carbohydrate**								
Jakobsen 2009 [42]	Pooled analysis of prospective cohort studies	11 studies (*n* = 344,696)	Range 4 to 10	Coronary events	5% of energy from SFA → total carbohydrate	↑	HR 1.07 (1.01–1.14)	Age; BMI; year survey completed; percentage of energy from MUFA, PUFA, *trans*-fat, protein and carbohydrates; energy intake; smoking; physical activity; education; alcohol intake; fiber intake; cholesterol intake; hypertension
				Coronary deaths	5% of energy from SFA → total carbohydrate	↔	HR 0.96 (0.82–1.13)	
Jakobsen 2010 [49]	Prospective cohort	53,644	Median 12	MI	5% of energy from SFA → total carbohydrates	↔	HR 1.04 (0.92–1.17)	Age, sex, BMI; percentage of energy from glycemic carbohydrates, proteins, MUFA, PUFA; energy intake; smoking; physical activity; education; alcohol consumer; intake of alcohol; hypertension
					5% of energy from SFA → carbohydrates with low-GI (median GI 82)	↔	HR 0.88 (0.72–1.07)	
					5% of energy from SFA → carbohydrates with medium-GI (median GI 88)	↔	HR 0.98 (0.80–1.21)	
					5% of energy from SFA → carbohydrates with high-GI (median GI 93)	↑	HR 1.33 (1.08–1.64)	
Guasch-Ferré 2015 [43] [PREDIMED]	Prospective cohort	7038	6	CVD	5% of energy from SFA→ total carbohydrate	↔	HR 0.83 (0.63–1.10)	Age; sex; BMI; intake of subtypes of fat, protein, and carbohydrates; energy intake; smoking; physical activity; education; alcohol intake; fiber intake; cholesterol intake; hypertension; intervention group; diabetes; hyper-cholesterolemia; family history of CHD; antihypertensive medication; oral antidiabetic agents; lipid lowering drugs
				All-cause death		↔	HR 1.04 (0.81–1.33)	
Li 2015 [44] [NHS; HPFS]	Prospective cohort	127,536	Range 24–30	CHD	5% of energy from SFA → whole grains	↓	HR 0.91 (0.85–0.98)	BMI, percentage of energy from protein; energy intake; smoking; physical activity; alcohol intake; cholesterol intake; hypertension at baseline; hypercholesterolemia at baseline; family history of myocardial infarction and diabetes; use of vitamins and aspirin
					5% of energy from SFA → refined starches/added sugar	↔	Not reported	
Zong 2016 [17] [NHS; HPFS]	Prospective cohort	115,782	NHS 25.8; HPFS 21.2	CHD	1% of energy from 12:0–18:0 SFA → whole grains	↓	HR 0.94 (0.91, 0.97)	Age; BMI; ethnicity; total energy; energy from *trans*-fat; energy from carbohydrates of non-whole grain sources; energy from non-plant sources; smoking status; physical activity; alcohol intake; family history of MI; menopausal status; postmenopausal hormone use; aspirin use; multivitamin use; baseline hypertension; baseline hypercholesterolemia; MUFA intake; PUFA intake; plant protein intake; intake of other SFA
Chen 2016 [41] [NHS; NHS II; HPFS]	Prospective cohort	134,327	NHS ≤ 32; NHS II ≤ 20; HPFS ≤ 24	CVD	5% of energy from dairy fat → carbohydrate from whole grains	↓	HR 0.72 (0.69–0.75)	Age, BMI, intake of protein; energy intake; smoking; physical activity; intake of fruit, vegetables, coffee; alcohol intake; baseline hypertension; baseline hyper-cholesterolemia; race; menopausal status and menopausal hormone use (NHS and NHS II); oral contraceptive use (NHS II only)
				CHD		↓	HR 0.66 (0.62–0.70)	
				Stroke		↓	HR 0.84 (0.78–0.91)	
				CVD	5% of energy from dairy fat → carbohydrate from refined starch and added sugar	↔	HR 0.97 (0.94–1.00)	
				CHD		↔	HR 0.96 (0.93–1.00)	
				Stroke		↔	HR 0.98 (0.94–1.03)	
Praagman 2016 [31] [EPIC-NL]	Prospective cohort	35,597	12	IHD	5% of energy from SFA → total carbohydrates	↑	HR (1.23 (1.09–1.40)	Age, sex, BMI, waist circumference; intake of carbohydrate, *cis*-MUFA, PUFA, *trans*-fat, animal protein and vegetable (per 5% of energy); energy intake (excluding alcohol); smoking, physical activity; education; alcohol intake; fiber intake (energy adjusted); cholesterol intake (energy adjusted); vitamin c (energy adjusted)
					5% of energy from SFA → carbohydrates with low GI (GI < 53)	↔	HR 1.14 (0.91–1.43)	
					5% of energy from SFA → carbohydrates with medium GI	↑	HR 1.35 (1.05–1.73)	
					5% of energy from SFA → carbohydrates with high GI (GI > 56)	↑	HR 1.27 (1.03–1.56)	
Hooper 2015 [46] Cochrane review	Meta-analysis of randomized controlled trials	15 studies (*n* > 59,000)	>2	CVD events	SFA → carbohydrate	↔	RR 0.93 (0.79–1.08)	Aggregate meta-analysis—no overall adjustment
**Substitution of Saturated Fat for Protein**								
Larsson 2012 [50]	Prospective cohort	34,670	Median 10.4	Stroke	5% of energy from SFA → protein	↓	13% lower risk (0–26%)	Age, BMI; intake of fat; energy intake; smoking status and smoking pack years; physical activity; education; alcohol intake; intake of cholesterol, calcium, fruits and vegetables; hypertension; diabetes; aspirin use; family history of myocardial infarction
Praagman 2016 [31] [EPIC-NL]	Prospective cohort	35,597	12	IHD	5% of energy from SFA → total protein	↑	HR 1.29 (1.08–1.54)	Age, sex, BMI, waist circumference; intake of carbohydrate, *cis*-MUFA, PUFA, *trans*-fat, animal protein and vegetable (per 5% of energy); energy intake (excluding alcohol); smoking, physical activity; education; alcohol intake; fiber intake (energy adjusted); cholesterol intake (energy adjusted); vitamin c (energy adjusted)
					5% of energy from SFA → animal protein	↑	HR 1.37 (1.14–1.65)	
					5% of energy from SFA → vegetable protein	↔	HR 0.81 (0.57–1.17)	
Zong 2016 [17] [NHS; HPFS]	Prospective cohort	115,782	NHS 25.8; HPFS 21.2	CHD	1% of energy from 12:0–18:0 SFA → plant protein	↓	HR 0.93 (0.89, 0.97)	Age; BMI; ethnicity; total energy; energy from *trans*-fat; energy from carbohydrates of non-whole grain sources; energy from non-plant sources; smoking status; physical activity; alcohol intake; family history of MI; menopausal status; postmenopausal hormone use; aspirin use; multivitamin use; baseline hypertension; baseline hypercholesterolemia; MUFA intake; whole grain intake; intake of other SFA
Hooper 2015 [46] Cochrane review	Meta-analysis of randomized controlled trials	15 studies (*n* > 59,000)	>2	CVD events	SFA → protein	↔	RR 0.98 (0.90–1.06)	Aggregate meta-analysis—no overall adjustment

**Table 2:** The effect of macronutrient substitutions of blood lipid levels as reported by Mensink 2016 [4].

Study	Design	*n*	Follow-Up Time	Outcome	Substitution	Result	Effect Size	Covariates Included in Analyses
**Substitution of Saturated Fat for MUFA**								
Mensink 2016 [4]	Systematic review and meta-analysis of randomized controlled trials	74 studies	Range 13–91 days	Total cholesterol	1% of energy from SFA → *cis*-MUFA	−0.046 mmol/L (−0.051 to−0.040; *p* < 0.001)	↓	No adjustment
		69 studies		LDL cholesterol		−0.042 mmol/L (−0.047 to −0.037; *p* < 0.001)	↓	
		68 studies		HDL cholesterol		−0.002 mmol/L (−0.00 to 0.000; *p* = 0.014))	↓	
		72 studies		Triglycerides		−0.004 mmol/L (−0.007 to −0.001; *p* = 0.022)	↓	
**Substitution of Saturated Fat for PUFA**								
Mensink 2016 [4]	Systematic review and meta-analysis of randomized controlled trials	74 studies	Range 13–91 days	Total cholesterol	1% of energy from SFA → *cis*-PUFA	−0.064 mmol/L (−0.070 to −0.058; *p* < 0.001)	↓	No adjustment
		69 studies		LDL cholesterol		−0.055 mmol/L (−0.061 to −0.050; p < 0.001)	↓	
		68 studies		HDL cholesterol		−0.005 mmol/L (−0.006 to −0.003; *p* < 0.001)	↓	
		72 studies		Triglycerides		−0.010 mmol/L (−0.014 to −0.007; *p* < 0.001)	↓	
**Substitution of Saturated Fat for Carbohydrate**								
Mensink 2016 [4]	Systematic review and meta-analysis of randomized controlled trials	74 studies	Range 13–91 days	Total cholesterol	1% of energy from SFA → carbohydrates	−0.041 mmol/L (−0.047 to −0.035; *p* < 0.001)	↓	No adjustment
		69 studies		LDL cholesterol		−0.033 mmol/L (−0.039 to −0.027; *p* < 0.001)	↓	
		68 studies		HDL cholesterol		−0.010 mmol/L (−0.012 to −0.008; *p* < 0.001)	↓	
		72 studies		Triglycerides		0.011 mmol/L (0.007 to 0.014; *p* = 0.842)	↔	

## Abbreviations

ACC	American College of Cardiology
AHA	American Heart Association
ALA	alpha linolenic acid (*n*-*3*)
AMDR	Acceptable Macronutrient Distribution Ranges
CAD	coronary artery disease
CHD	coronary heart disease
CVD	cardiovascular disease
DGAs	Dietary Guidelines for Americans
DHA	docosahexaenoic acid
EPA	eicosapentaenoic acid
EPIC	European Prospective Investigation into Cancer and Nutrition
GI	glycemic index
GL	glycemic load
HDL	high-density lipoprotein
HPFS	Health Professionals Follow-Up Study
hs-CRP	high sensitivity C-reactive protein
IHD	ischemic heart disease
LCFA	long chain fatty acids
LDL	low-density lipoprotein
MCFA	medium chain fatty acids
MUFA	monounsaturated fatty acids
MI	myocardial infarction
NHS	Nurse’s Health Study
PREDIMED	Prevención con Dieta Mediterránea
PUFA	polyunsaturated fatty acids
SCD	sudden cardiac death
SFA	saturated fatty acids
